# Acetylated-PPARγ expression is regulated by different *P53* genotypes associated with the adipogenic differentiation of polyploid giant cancer cells with daughter cells

**DOI:** 10.20892/j.issn.2095-3941.2022.0432

**Published:** 2023-01-12

**Authors:** Kexin Zhang, Xiaohui Yang, Minying Zheng, Yidi Ning, Shiwu Zhang

**Affiliations:** 1 Tianjin Union Medical Center, Tianjin 300122, China; 2 Nankai University School of Medicine, Nankai University, Tianjin 300071, China

**Keywords:** PGCCs, adipogenic differentiation, PPARγ, post-translational modification, *P53* genotype

## Abstract

**Objective:**

Polyploid giant cancer cells (PGCCs) with daughter cells express epithelial–mesenchymal transition (EMT)-associated proteins. Highly malignant tumor cells with EMT properties can transdifferentiate into mature tumor cells. In this study, we elucidated the potential for, and underlying mechanism of, adipogenic differentiation of PGCCs with daughter cells (PDCs).

**Methods:**

Cobalt chloride was used to induce PGCC formation in HEY (wild-type *P53*) and MDA-MB-231 (mutant *P53*) cells; these cells were then cultured in adipogenic differentiation medium. Oil red O staining was used to confirm adipogenic differentiation, and the cell cycle was detected with flow cytometry. The expression of adipogenic differentiation-associated proteins and P300 histone acetyltransferase activity were compared before and after adipogenic differentiation. Animal xenograft models were used to confirm the adipogenic differentiation of PDCs.

**Results:**

PDCs transdifferentiated into functional adipocytes. Two different cell cycle distributions were observed in PDCs after adipogenic differentiation. The expression levels of PPARγ, Ace-PPARγ, and Ace-P53 were higher in PDCs after adipogenic differentiation than in cells before adipogenic differentiation. Ace-PPARγ and FABP4 expression increased in HEY cells and decreased in MDA-MB-231 PDCs after *p53* knockdown. A485 treatment increased Ace-P53, Ace-PPARγ, and FABP4 expression in HEY PDCs by inhibiting SUMOylation of P53. In MDA-MB-231 PDCs, A485 treatment decreased Ace-P53, Ace-PPARγ, and FABP4 expression. Animal experiments also confirmed the adipogenic differentiation of PDCs.

**Conclusions:**

Acetylation of P53 and PPARγ plays an important role in the adipogenic differentiation of PDCs.

## Introduction

The adipogenic differentiation of human mesenchymal stem cells is typically induced with a cocktail of 3-isobutyl-1-methylxanthine (IBMX), dexamethasone, and insulin^1^. Adipocytes may be derived from not only preadipocytes and pluripotent mesenchymal stem cells, but also cancer stem cells (CSCs). One treatment for malignant tumors is induced differentiation therapy, which involves treatment with chemicals that promote the differentiation of malignant cells into normal cells. Well-differentiated and dedifferentiated liposarcoma cells can also be differentiated into adipocytes with dexamethasone, indomethacin, insulin, and IBMX. These compounds induce adipogenesis by upregulating the transcription and translation of genes involved in maintaining cancer cell stemness and adipogenic differentiation^2^. Ishay-Ronen et al.^3^ have reported that combinatorial treatment with MEK inhibitors and the antidiabetic drug rosiglitazone induces the conversion of invasive and metastatic breast cancer cells into adipocytes, thereby repressing primary tumor cell invasion and metastasis in breast cancer.

We have reported that cobalt chloride (CoCl_2_), radiation, and chemotherapy drugs induce the formation of polyploid giant cancer cells (PGCCs), and daughter cells derived from PGCCs *via* asymmetric division (budding and bursting) have strong invasion and infiltration abilities^4–6^. PGCCs with daughter cells (PDCs) have CSC properties and express multiple normal and CSC markers, including CD44, CD133, Nanog, and SOX-2^7,8^. Additionally, PDCs express epithelial–mesenchymal transition (EMT)-associated proteins, including high expression of mesenchymal markers and low expression of epithelial markers^9,10^. EMT is a de-differentiation process that is known to enhance cellular plasticity, and can be exploited therapeutically through transdifferentiation into post-mitotic and functional cells^3^. PDCs are necessary for cancer dissemination, but can be directly targeted and inhibited through a transdifferentiation approach. We previously reported that PDCs can be induced to differentiate into multiple benign lineages, such as adipocytes, bone, and cartilage^6,11,12^. However, the underlying molecular mechanism remains unclear. In this study, CoCl_2_-treated HEY and MDA-MB-231 PDCs were cultured in adipogenic differentiation medium and induced to differentiate into adipocytes. We assessed the critical transcription factors and post-translational modifications (PTMs) in adipogenic differentiation in HEY and MDA-MB-231 PDCs, including PPARγ, which has recently been demonstrated to play an important role in adipogenic differentiation^13^. In addition, recent studies have reported that fibroblast-derived cancer cells with *p53* gene deletion can be induced to differentiate into adipocytes^14^. After adipogenic differentiation, Ace-P53 expression levels are regulated by different *P53* genotypes. Fatty acid binding protein 4 (FABP4) is a member of the FABP family that is abundantly expressed in adipocytes. Expression of FABP4, a marker of successful adipogenic differentiation of PDCs, is associated with the acetylation of PPARγ and different *P53* genotypes. Understanding the complex regulatory mechanisms in malignant tumors with EMT properties that can transdifferentiate into mature tumor cells may lead to the development of new therapies for solid tumors.

## Materials and methods

### Cell culture

HEY and MDA-MB-231 cell lines were obtained from the American Type Culture Collection (USA), HEY cells were cultured in 1640 medium (Gibco, Thermo Fisher Scientific, Suzhou, China). MDA-MB-231 cells were cultured in Dulbecco’s modified Eagle’s medium (Sigma-Aldrich, St. Louis, MO, USA) supplemented with 10% fetal bovine serum (FBS) (Gibco, Life Technologies, New Zealand) and 1% penicillin/streptomycin (Gibco, Life Technologies, USA). Medium supplemented with serum and antibiotics was defined as complete medium. Cells cultured in complete medium were maintained in a humidified atmosphere at 37 °C and 5% CO_2_.

### Induction of PGCCs

Our previous research has described the method of CoCl_2_ induction of PGCC formation^6^. Briefly, HEY and MDA-MB-231 cells were cultured in T25 cell flasks containing complete medium until they reached 80%–90% confluency. Cells were treated with 450 μM CoCl_2_ (Sigma-Aldrich) for 48–72 h depending on their resistance to hypoxia. Most regular-sized cells died after CoCl_2_ treatment, and only several scattered PGCCs survived. Surviving PGCCs began to produce daughter cells *via* asymmetric division after 10–15 days. CoCl_2_ treatment was repeated 3 times to obtain sufficient PDCs for subsequent experiments.

### Adipogenic differentiation of cancer cells

**Figure 1:**
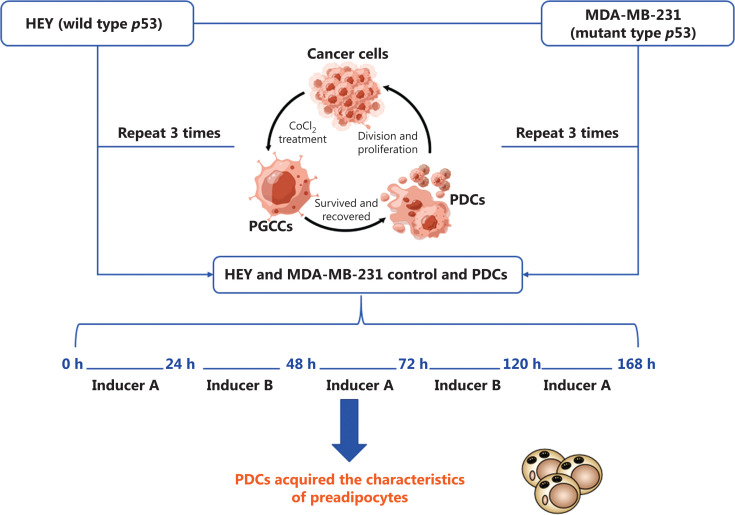
Workflow of PGCC induction and adipogenic differentiation in HEY and MDA-MB-231 control cells and PDCs.

Control cells (2.5 × 10^5^) and PDCs were seeded into 6-well plates and cultured in complete medium until they reached 70%–80% confluence. The medium was replaced with differentiation medium A for 24 h (StemPro Adipogenic Differentiation Kit; Cyagen Biosciences, Suzhou, China, containing 10% FBS, 0.5 mM IBMX, 4 μM insulin, 1 μM dexamethasone, and 10 mM rosiglitazone), then was replaced with differentiation medium B and incubated another 48 h (α-MEM containing 10% FBS and 4 μM insulin). The cells were repeatedly cultured in media A and B and harvested for subsequent experiments. The workflow of PGCC induction and adipogenic differentiation in HEY and MDA-MB-231 control cells and PDCs is shown in **Figure 1**.

### Oil red O (ORO) staining

ORO staining was performed according to the manufacturer’s instructions. HEY and MDA-MB-231 control cells and PDCs before and after adipogenic differentiation were fixed with 4% paraformaldehyde for 24 h at 25 °C and were then incubated with fresh ORO (Solarbio, Beijing, China) for 15 min. ORO-stained intracellular lipids were quantified on the basis of optical density at 510 nm with a microplate reader (BioTek, USA).

### Western blot (WB)

Proteins were fully denatured by boiling at 100 °C for 10 min. After the concentrations were determined, the proteins were separated on a 10% sodium dodecyl sulfate-polyacrylamide gel and transferred to a polyvinylidene fluoride membrane (Beyotime, Haimen, China). The membrane was blocked with 5% skim milk at room temperature for 2 h, then incubated with primary antibodies. Detailed antibody information is provided in **Supplementary Table S1**. All WB assays were performed in triplicate.

### Flow cytometry

To examine the DNA content and cell cycle status, we harvested control cells and PDCs before and after adipogenic differentiation. The cell pellets were fixed with 75% ethanol at 20 °C for 12 h and then permeabilized with 0.1% Triton X-100 at 26 °C for 20 min. After 30 min of RNase treatment at 37 °C, the cells were incubated with propidium iodide (50 μg/mL) at 25 °C for 15 min. The cell cycle distribution was analyzed with flow cytometry (BD FACSCalibur™, BD Biosciences). The proportions of cells in G1, S, and G2 phases were quantified with BD CellQuestä version 5.1 (BD Biosciences).

### Quantitative real-time PCR (qPCR)

Total RNA was extracted with the TRIzol/chloroform method. RNA was reverse-transcribed with a high-capacity RNA to cDNA kit (TIAGEN, KR116, Beijing, China). qPCR was performed on a Roche LightCycler 480 Real-Time PCR System (Roche). The qPCR reaction system included 2 μL of cDNA diluted in nuclease-free water, 50 ng of total RNA, 25 μL of Universal PCR Master Mix (CWBIO, 0957), and 1 μL of 10 μM forward primer. Nuclease-free water was used to dilute the reverse primer to a final volume of 50 μL, and β-actin was used as the reference gene for the quality and quantity of cDNA.

### Transient siRNA transfection

*P53* was knocked down through transient siRNA transfection. The siRNAs were synthesized by GenePharma (Shanghai, China) and included 3 siRNA interference sequences: GAPDH, 1 negative control (NC) sequence, and 1 mock control (MC). Three *P53* transfection sequences (339, 886, and 985) were used to knock down *P53* (**Supplementary Table S2**). Control cells and PDCs grown in 6-well plates at 60%–80% confluence were transfected with NC siRNA, *P53*-siRNA, MC, and GAPDH-siRNA with Lipofectamine 2000 (Invitrogen, Carlsbad, CA, USA) and 1× Opti-MEM (Gibco, USA) according to the manufacturers’ instructions [siRNA:lipo = 20:1 (pmol:μL)]. After transfection for 48 h, WB was performed to estimate the knockdown efficiency.

### P300 histone acetyltransferase activity assays

HEY and MDA-MB-231 cells were treated with sodium butyrate for 48 h. Nuclear extracts were harvested as described above and assayed. Briefly, each nuclear extract was incubated with 100 μM acetyl-CoA and 1× HAT assay buffer on a histone H3-precoated enzyme-linked immunosorbent assay plate for 30 min. P300 transfers the acetyl group of acetyl-CoA to histone peptides, thus resulting in the formation of sulfhydryl-CoA, which reacts with Ellman’s reagent, thereby altering the absorbance peak. Acetylated histones were detected with a HAT assay kit after several washes with PBS, according to the manufacturer’s protocol. Fluorescence intensity was detected with a multifunctional microplate reader (BioTek) at 412 nm. HAT activity was calculated with the following equation: [(sample reading-background reading) × 0.1 (system capacity; mL) × sample dilution factor] ÷ [0.01 (sample capacity; mL) × 13.6 (millimolar absorbance coefficient) × 15 (min)] = unit/mL ÷ (sample protein concentration) mg/mL = unit/mg.

### A485 inhibitor treatment

A485 is a potent and selective catalytic inhibitor of P300/CBP^15^. Control cells and PDCs before and after adipogenic differentiation were cultured in 6-well plates until they reached 80% confluence. Each well was treated with 5 μM A485 (Selleck, USA) for 72 h.

### Cell counting kit-8 (CCK8) assays

Cell viability was evaluated with CCK8 assays. Control cells and PDCs before and after adipogenic differentiation were seeded at a density of 2,000 cells per well in 96-well plates (3 replicate wells per group) and incubated for 12 h. Wells containing medium alone were used as controls. The cells were treated with different concentrations of A485 and cultured for various time intervals. After treatment, 10 μL CCK8 (Dojindo, Japan) was added to each well and incubated for 2 h. The optical density was measured at 450 nm with a Bio-Rad microplate reader, and the final value was calculated with the average value of the readings after subtraction of the average value of the control.

### Co-immunoprecipitation (Co-IP)

Co-IP was performed with a Pierce Classic Magnetic IP/Co-IP Kit (cat. No. 87787, Thermo Fisher Scientific, USA). Control cells and PDCs before and after adipogenic differentiation were lysed with IP Lysis Buffer (Thermo Fisher Scientific) containing Halt protease and phosphatase inhibitor cocktail (1:100 dilution). After centrifugation, the supernatant was incubated with antibodies at 4 °C overnight. Rabbit IgG (Beyotime, Shanghai, China) was used as the NC. Prewashed Protein A/G agarose beads (Thermo Fisher Scientific) were added to each IP tube and incubated. The immunoprecipitates were examined with WB.

### Immunocytochemical (ICC) and immunohistochemical (IHC) staining

For ICC, cells grown on coverslips were fixed with cold methanol for 30 min. After treatment with 0.3% endogenous peroxidase inhibitor (Zhongshan Inc., Beijing, China), cells were incubated with goat serum (Zhongshan Inc.) to block non-specific protein binding. The cells on coverslips were then incubated with primary antibodies, biotinylated goat anti-mouse/rabbit IgG (Zhongshan Inc.), and horseradish peroxidase-labeled streptomycin (Zhongshan Inc.). Paraffin-embedded tissue sections were deparaffinized in xylene for IHC. Antigen retrieval was performed by incubation of sections with citrate buffer (OriGene, Wuxi, China). The sections were then incubated with primary antibodies and biotinylated goat anti-rabbit IgG. The signal was detected with a labeled streptavidin-biotin system in the presence of the chromogen 3,3-diaminobenzidine or alkaline phosphatase.

### Wound-healing assays

Control cells and PDCs before and after adipogenic differentiation were seeded into 12-well plates (1 × 10^5^ cells per well, 3 replicate wells per group) and cultured until they reached 100% confluence. Sterile pipette tips were then used to uniformly scratch the monolayer of cells vertically to form wound tracks. After being rinsed with PBS to remove floating cells, the cells were cultured in serum-free medium. Cell migration was evaluated by imaging of the wound area at 0, 12, and, 24 h for HEY cells and at 0, 16, and, 32 h for MDA-MB-231 cells at the same scratch position. The migration area was outlined in ImageJ, and the wound-healing index was calculated with the following formula: [(wound area at 0 h) − (wound area at indicated time)]/(wound area at 0 h). A high score indicated strong migration ability.

### Cell migration and invasion assays

Control cells and PDCs before and after adipogenic differentiation were washed 3 times with serum-free medium and counted with an automated cell counter (Invitrogen). Cell migration and invasion were assessed with Transwell migration and invasion assays (8 μm; Corning Inc.), respectively. For the migration assays, 5 × 10^4^ cells per insert, resuspended in 200 μL serum-free medium, were seeded in the upper chamber. For the invasion assay, 2 × 10^5^ cells per insert, resuspended in 200 μL serum-free medium, were seeded in the upper chamber coated with Matrigel. Medium containing 20% FBS was added to the lower chamber, and the 24-well plates were incubated for an additional 12–24 h at 37 °C. The cells were then fixed in methanol for 30 min and stained with 0.1% crystal violet for 30 min. The migration and invasion abilities were assessed by counting the number of cells per field. Images were acquired at 100× magnification, and cells in at least 5 different fields were counted. Three independent experiments were performed.

### Clone formation assays

Control cells and PDCs were counted before and after adipogenic differentiation, respectively. Cell suspensions (2 mL/well) containing 30, 60, and 120 cells were cultured in 12-well plates, and the plates were incubated for at least 2 weeks at 37 °C (when white cell clones were visible). Cell clones were washed with PBS and fixed with cold methanol for 30 min. After staining with 0.1% crystal violet for 30 min, the number of cell clone groups per well was counted under a microscope (the number of cells in a single clone was >50), and the efficiency of colony formation was calculated with the following formula: formation efficiency = (number of clones/number of cells inoculated).

### Animal experiments

Forty 4- to 6-week-old female BALB/c NU/NU nude mice (20 for HEY and 20 for MDA-MB-231) were obtained from Beijing Weitonglihua Co. Ltd. Twenty nude mice for each cell line were divided into 4 groups (5 mice per group), and mice in each group were injected with 200 μL (1 × 10^6^ cells) of the tumor cell suspension containing one of the following: (i) control cells (CC), (ii) control cells after adipogenic differentiation (CA), (iii) PDCs (PC), or (iv) PDCs after adipogenic differentiation (PA). When the diameter of the tumor reached 0.3 cm, the size of the tumor was measured every other day. The animals were euthanized on day 30 for HEY cells and day 45 for MDA-MB-231 cells after inoculation. Paraffin-embedded tumor tissues were used for hematoxylin and eosin (H&E) and IHC staining, and fresh tumor tissues were used for WB analysis. The animal experiments were approved and supported by the Institutional Animal Care and Use Committee of the Tianjin Union Medical Center (Approval No. 2022B37).

### H&E staining

Tumor tissues were fixed in formalin for 24 h at room temperature and embedded in paraffin, and 4 μm-thick sections were prepared. The tissue sections were subsequently deparaffinized in xylene for 12 h at 75 °C and rehydrated with a descending ethanol series. Sections were stained with 0.2% hematoxylin (Baso, Guangzhou, China) at room temperature for 1 min and 0.5% eosin for 2 min. After staining, the sections were dehydrated and mounted on coverslips.

### Statistical analysis

Statistical analyses and graphs were generated in SPSS 22 software (SPSS Inc., Chicago, USA) and GraphPad Prism software. Statistical analyses were performed with ANOVA and unpaired *t*-test. The number of animals used in each experiment is indicated in the figure legends. The *in vivo* statistical analysis was performed with the Kruskal–Wallis test and corrected for multiple comparisons. The threshold for statistical significance was set at *P* < 0.05. *P*-values represent comparisons with each control (***P < 0.001, **P < 0.005, and *P < 0.05).

## Results

### CoCl_2_ induces the formation of PGCCs, and PDCs can differentiate into adipocytes

**Figure 2:**
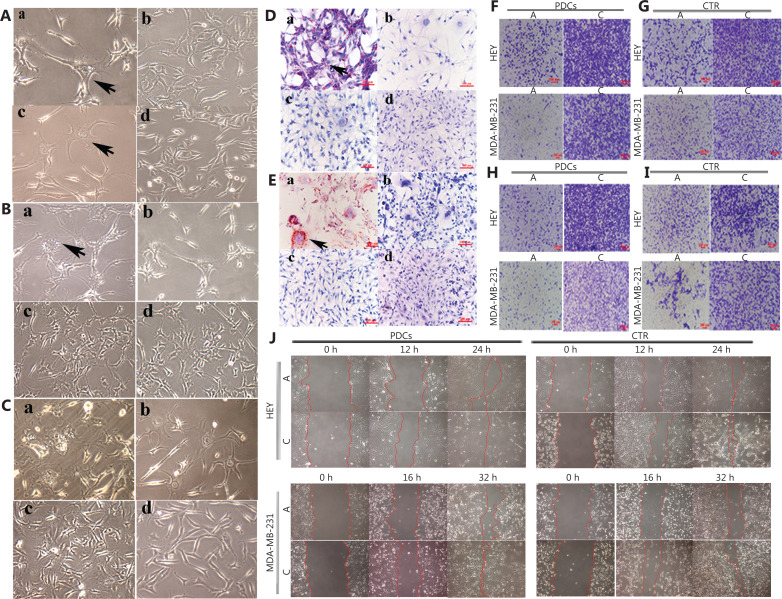
Formation of PDCs and adipogenic differentiation. (A) HEY and MDA-MB-231 PDCs (100×). (a) HEY PDCs. (b) HEY control cells. (c) MDA-MB-231 PDCs. (d) MDA-MB-231 control cells. (B) Adipogenic differentiation of HEY control cells and PDCs (100×). (a) HEY PDCs after adipogenic differentiation. (b) HEY PDCs. (c) HEY control cells after adipogenic differentiation. (d) HEY control cells. (C) Adipogenic differentiation of MDA-MB-231 control cells and PDCs (100×). (a) MDA-MB-231 PDCs after adipogenic differentiation. (b) MDA-MB-231 PDCs. (c) MDA-MB-231 control cells after adipogenic differentiation. (d) MDA-MB-231 control cells. (D) Oil red O staining of HEY cells (100×). (a) PDCs after adipogenic differentiation. (b) PDCs. (c) Control cells after adipogenic differentiation. (d) Control cells. (E) Oil red O staining of MDA-MB-231 (100×). (a) PDCs after adipogenic differentiation (b) PDCs. (c) Control cells after adipogenic differentiation. (d) Control cells. (F) Transwell assay showing invasion ability in HEY and MDA-MB-231 PDCs before and after differentiation (100×). (G) Transwell assay showing invasion ability in HEY and MDA-MB-231 control cells before and after differentiation (100×). (H) Transwell assay showing migration ability in HEY and MDA-MB-231 PDCs before and after differentiation (100×). (I) Transwell assay showing migration ability in HEY and MDA-MB-231 control cells before and after differentiation (100×). (J) Wound-healing assays for HEY and MDA-MB-231 PDCs and control cells, with or without adipogenic differentiation.

HEY and MDA-MB-231 cells were treated with CoCl_2_ (450 μM for 48 h). Most regular-sized tumor cells died, and only a small number of cells with large nuclei (PGCCs) survived (**Figure 2Aa and 2Ac**). After culturing in medium without CoCl_2_ for 10–15 days, PGCCs produced daughter cells *via* asymmetric division. The PGCCs were 3 times larger than the regular control cells (**Figure 2Ab and 2Ad**). HEY and MDA-MB-231 control cells and PDCs were cultured in adipogenic differentiation medium containing rosiglitazone, dexamethasone, IBMX, and insulin for 7 days. Comparison of the cells cultured in adipogenic differentiation medium indicated that PDCs had more vacuoles in their cytoplasm (**Figure 2Ba and 2Ca**) than the control cells (**Figure 2Bc and 2Cc**). ORO staining revealed the presence of more red lipid droplets in the PDCs (**Figure 2Da, 2Db and 2Ea, 2Eb**) than the controls (**Figure 2Dc, 2Dd and 2Ec, 2Ed**). ORO staining also revealed greater intracellular lipid accumulation in PDCs (**Supplementary Figure S1Aa and S1Ac**) than the controls (**Supplementary Figure S1Ab and S1Ad**) when the cells were cultured in adipogenic differentiation medium.

### Adipocyte differentiation decreases the invasion, metastasis and proliferation of control cells and PDCs

Cell invasion assays were performed with Matrigel-coated Transwell inserts. The invasive (**Figure 2F and 2G**) and migratory abilities (**Figure 2H and 2I**) of cells cultured in adipogenic differentiation medium were lower than those of cells cultured in complete medium. PDCs cultured in adipogenic differentiation medium had the lowest number of invasive and migratory cells among all cell groups (**Supplementary Figure S1B and S1C**). Migration ability was also measured with wound-healing assays in HEY and MDA-MB-231 control cells and PDCs before and after adipogenic differentiation. The migration ability of PDCs was higher than that of control cells. After adipogenic differentiation, migration ability decreased in both control cells and PDCs (**Figure 2J**). Significant differences were observed in wound-healing indices with or without adipogenic differentiation in the HEY control cells and PDCs at 12 and 24 h, and in the MDA-MB-231 control cells and PDCs at 16 and 32 h (**Supplementary Figure S1D**). To detect cell proliferative ability, we performed plate cloning assays. The number of clones formed in the HEY and MDA-MB-231 PDCs after adipogenic differentiation was significantly lower than that in the corresponding cells without adipogenic differentiation (**Supplementary Figure S1E and S1F**).

### Adipogenic differentiation-associated protein expression in adipogenic differentiation of PDCs

**Figure 3:**
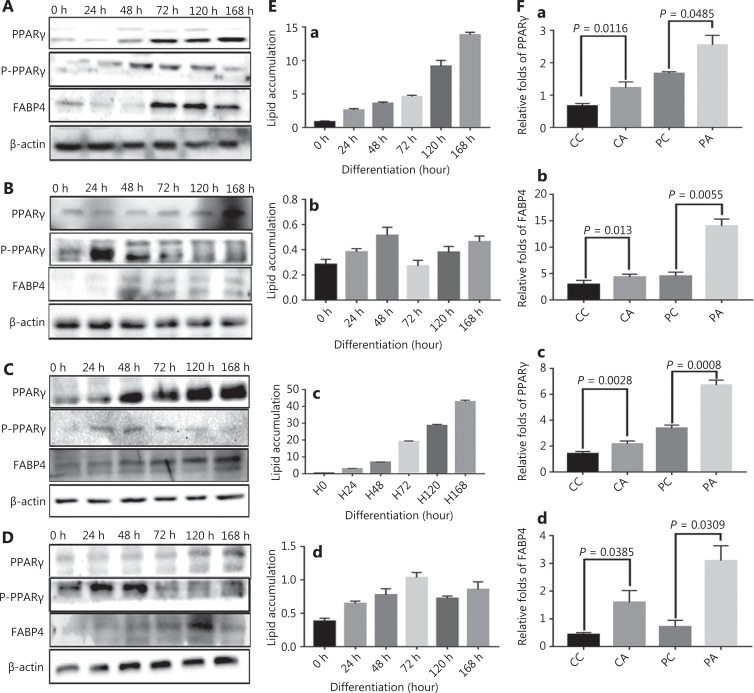

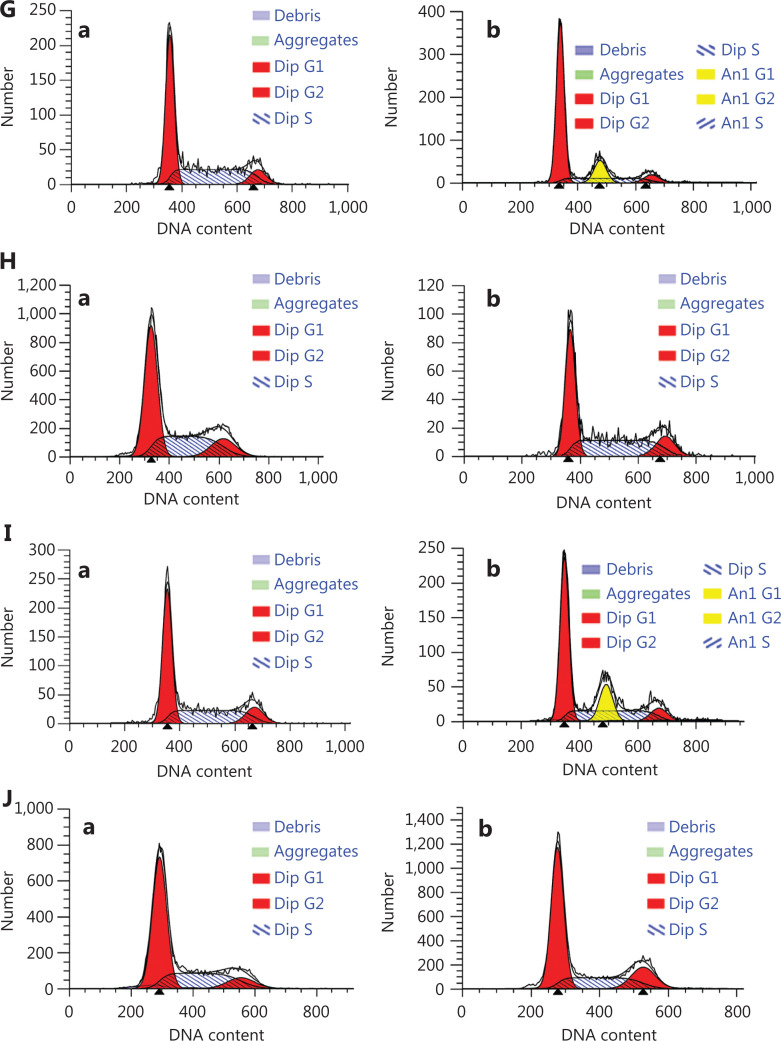
Time-dependent expression of PPARγ after adipogenic differentiation. Western blot showing the time-dependent expression of PPARγ, phospho-PPARγ (Ser112), and FABP4 in (A) HEY PDCs, (B) HEY control cells, (C) MDA-MB-231 PDCs, and (D) MDA-MB-231 control cells. (E) Oil red O-stained lipid droplets were extracted for quantification of time-dependent lipid accumulation in cells after adipogenic differentiation. (a) HEY PDCs. (b) HEY control cells. (c) MDA-MB-231 PDCs. (d) MDA-MB-231 control cells. (F) Real-time quantitative PCR confirmation of PPARγ, and FABP4 expression in CC, CA, PC, and PA. (a) PPARγ in HEY. (b) FABP4 in HEY. (c) PPARγ in MDA-MB-231. (d) FABP4 in MDA-MB-231. (G) Flow cytometry evaluation of the cell cycle in (a) HEY PDCs and (b) HEY PDCs after adipogenic differentiation. (H) Flow cytometry evaluations of the cell cycle in (a) HEY control cells and (b) HEY control cells after adipogenic differentiation. (I) Flow cytometry evaluation of the cell cycle in (a) MDA-MB231 PDCs and (b) MDA-MB-231 PDCs after adipogenic differentiation. (J) Flow cytometry evaluations of the cell cycle in (a) MDA-MB-231 control cells and (b) MDA-MB-231 control cells after adipogenic differentiation.

In HEY and MDA-MB-231 PDCs, the expression of PPARγ and FABP4 gradually increased with increasing adipogenic differentiation time, whereas that of phosphor-PPARγ (Ser112) gradually decreased (**Figure 3A and 3C**). No clear change was observed in the expression of PPARγ and FABP4 in HEY and MDA-MB-231 control cells with increasing adipogenic differentiation time (**Figure 3B and 3D**). Statistical analysis indicated that intracellular lipid accumulation in PDCs increased with increasing culture time in adipogenic differentiation medium (**Figure 3Ea and 3Ec**); however, analysis of intracellular lipid accumulation did not reveal any significant change in the control cells (**Figure 3Eb and 3Ed**). The results of qPCR indicated that the mRNA expression levels of PPARγ (for HEY: control *vs.* control after adipogenic differentiation, *P* = 0.0116; PDCs *vs.* PDCs after adipogenic differentiation, *P* = 0.0485; for MDA-MB-231: control *vs.* control after adipogenic differentiation, *P* = 0.0028; PDCs *vs.* PDCs after adipogenic differentiation, *P* = 0.0008) and FABP4 (for HEY: control *vs.* control after adipogenic differentiation, *P* = 0.013; PDCs *vs.* PDCs after adipogenic differentiation, *P* = 0.0055; for MDA-MB-231: control *vs.* control after adipogenic differentiation, *P* = 0.0385; PDCs *vs*. PDCs after adipogenic differentiation, *P* = 0.0309) were significantly higher in HEY and MDA-MB-231 control cells and PDCs with adipogenic differentiation than in those without adipogenic differentiation (**Figure 3F**).

### Flow cytometric analysis and cell cycle-associated protein expression in control cells and PDCs before and after adipogenic differentiation

**Figure 4:**
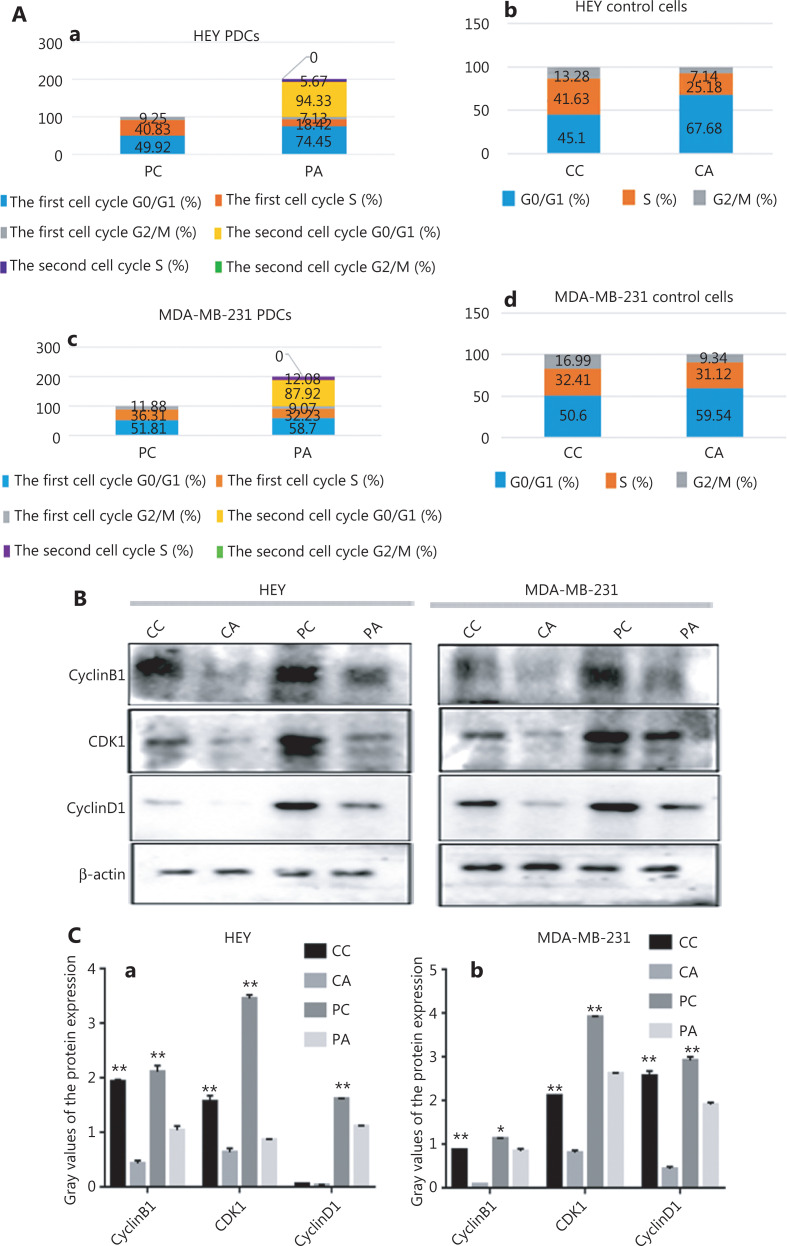

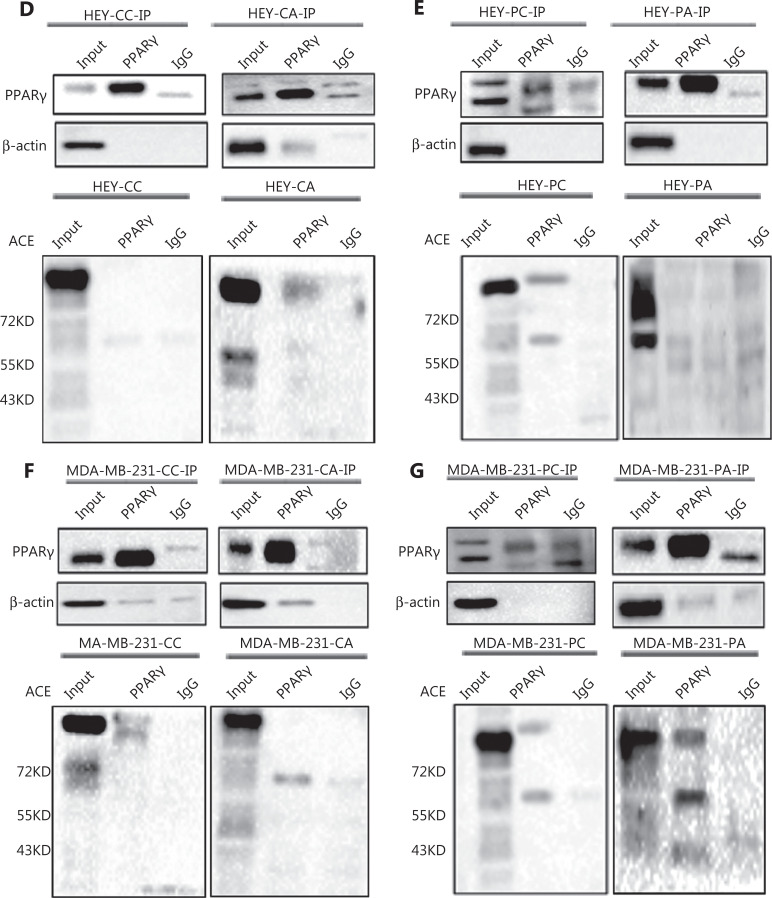
The cell cycle before and after adipogenic differentiation. (A) Columnar percentage plot showing the ratios of cells in G1, S, and G2 stages of the cell cycle in (a) HEY PDCs, (b) HEY control, (c) MDA-MB-231 PDCs, and (d) MDA-MB-231 control cells before and after adipogenic differentiation. (B) Results of WB showing the expression of cyclin B1, CDK1, and cyclin D1 in HEY and MDA-MB-231 control cells and PDCs before and after adipogenic differentiation. (C) Histograms showing cyclin B1, CDK1, and cyclin D1 expression in (a) HEY and (b) MDA-MB-231 control cells and PDCs before and after adipogenic differentiation (*P<0.05, **P<0.01). (D) Total lysates of HEY control cells before and after adipogenic differentiation, immunoprecipitated with anti-PPARγ and immunoblotted with anti-ACE. (E) Total lysates of HEY PDCs before and after adipogenic differentiation, immunoprecipitated with anti-PPARγ and immunoblotted with anti-ACE. (F) Total lysates of MDA-MB-231 control cells before and after adipogenic differentiation treatment, immunoprecipitated with anti-PPARγ and immunoblotted with anti-ACE. (G) Total lysates of MDA-MB-231 PDCs before and after adipogenic differentiation, immunoprecipitated with anti-PPARγ and immunoblotted with anti-ACE.

HEY and MDA-MB-231 cells with or without CoCl_2_ treatment, cultured in adipogenic differentiation medium for 168 h, were subjected to DNA content and cell cycle analyses. After the HEY and MDA-MB-231 cells were treated with CoCl_2_, PDCs had a greater number of cells arrested in G2/M phase (HEY PGCCs: 9.25%; MDA-MB-231 PGCCs: 11.88%) than the control cells (HEY control cells: 7.14%; MDA-MB-231 control cells: 9.34%). This finding indicated that CoCl_2_ induced cell cycle arrest at G2/M phase in HEY and MDA-MB-231 PDCs. However, flow cytometric analysis revealed 2 different cell cycle distributions (yellow peak) in HEY (**Figure 3Gb**) and MDA-MB-231 (**Figure 3Ib**) PDCs 7 days after adipogenic differentiation than in PDCs (**Figure 3Ga and 3Ia**) before adipogenic differentiation. No discernible difference was observed in the cell cycle between HEY (**Figure 3Ha and 3Hb**) and MDA-MB-231 (**Figure 3Ja and 3Jb**) control cells before and after adipogenic differentiation. In the first distribution, 74.45% and 58.7% of the HEY and MDA-MB-231 PDCs, respectively, were arrested in G1 phase after adipogenic differentiation, and 49.92% and 51.81% of the HEY and MDA-MB-231 PDCs, respectively, were arrested in G1 phase without adipogenic differentiation; for the second distribution, 94.33% and 87.92% of the HEY and MDA-MB-231 PDCs, respectively, were arrested in G1 phase after adipogenic differentiation (**Figure 4Aa and 4Ac**). The proportions of cells in S and G0/G1 phases in the HEY and MDA-MB-231 control cells increased after adipogenic differentiation (HEY control cells: 67.68%; MDA-MB-231 control cells: 59.54%; HEY control cells: 45.1%; MDA-MB-231 control cells: 50.6%) (**Figure 4Ab and 4Ad**). Additionally, compared with those without adipogenic differentiation, the expression levels of cyclin B1, CDK1, and cyclin D1 decreased in HEY and MDA-MB-231 PDCs after adipogenic differentiation (**Figure 4B**). The differences in cyclin B1, CDK1, and cyclin D1 expression in the cells before and after adipogenic differentiation were statistically significant (*P < 0.05, **P < 0.01) (**Figure 4C**).

### Acetylation modification of PPARγ in HEY and MDA-MB-231 after adipogenic differentiation

**Figure 5:**
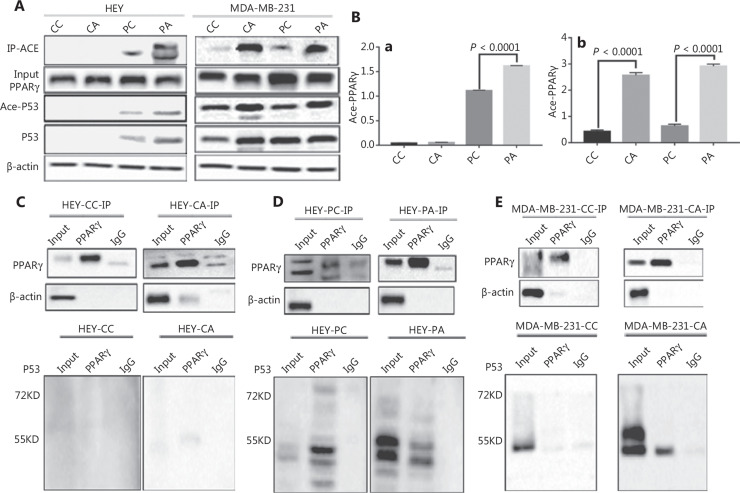

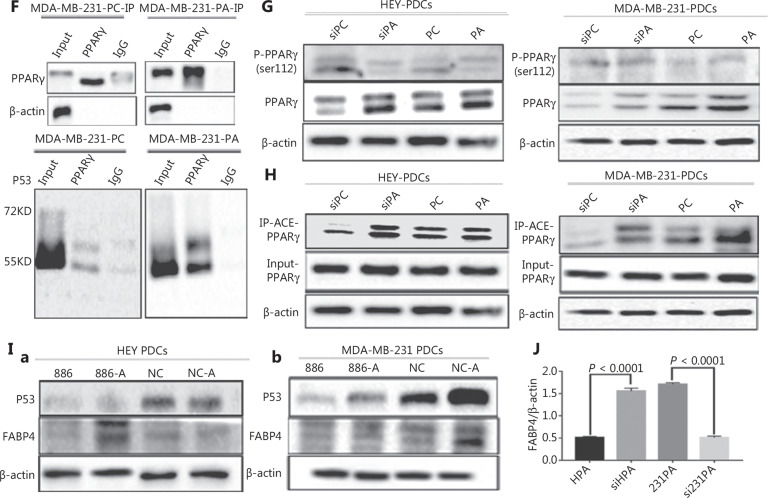
Ace-PPARγ expression before and after adipogenic differentiation. (A) Expression of Ace-PPARγ, Ace-p53, and P53 in HEY and MDA-MB-231 control cells and PDCs before and after adipogenic differentiation. (B) Histogram of Ace-PPARγ expression in (a) HEY and (b) MDA-MB-231 control cells and PDCs, before and after adipogenic differentiation. Total lysates of (C) HEY and (D) HEY PDCs (E) MDA-MB-231 control cells, and (F) MDA-MB-231 PDCs before and after adipogenic differentiation, immunoprecipitated with anti-PPARγ and immunoblotted with anti-P53. (G) Western blot showing the expression of phospho-PPARγ (Ser112) and PPARγ in HEY and MDA-MB-231 PDCs before and after adipogenic differentiation and siRNA-*P53* transfection. (H) Expression of Ace-PPARγ in HEY and MDA-MB-231 PDCs with or without adipogenic differentiation after *P53* knockdown. (I) Western blot showing the expression of P53 and FABP4 in (a) HEY PDCs and (b) MDA-MB-231 PDCs, with or without adipogenic differentiation after siRNA-p53 transfection. (J) Histograms showing the expression of FABP4 in HEY and MDA-MB-231 PDCs with or without adipogenic differentiation after *P53* knockdown.

As described above, the expression of total PPARγ gradually increased with increasing adipogenic differentiation time, whereas that of phospho-PPARγ (Ser112) gradually decreased in HEY and MDA-MB-231 PDCs. To determine other PTMs of PPARγ, we performed a combination of IP and WB to investigate the expression of Ace-PPARγ in HEY and MDA-MB-231 control cells and PDCs before and after adipogenic differentiation. As shown in **Figure 4D and 4G**, PPARγ and pan-lysine acetylation were detected in HEY and MDA-MB-231 control cells and PDCs before and after adipogenic differentiation. The results confirmed that acetylation modification of PPARγ occurred in HEY and MDA-MB-231 PDCs after adipogenic differentiation. WB results confirmed that the expression levels of Ace-PPARγ and Ace-P53 were higher in HEY and MDA-MB-231 PDCs after adipogenic differentiation than in PDCs before adipogenic differentiation (**Figure 5A**), and the difference in Ace-PPARγ expression was statistically significant (for HEY: PDCs *vs.* PDCs after adipogenic differentiation, *P* = 0.000; for MDA-MB-231: control *vs.* control after adipogenic differentiation, *P* = 0.0000; PDCs *vs.* PDCs after adipogenic differentiation *P* = 0.0000) (**Figure 5B**).

### PPARγ interacts with P53, and P53 regulates total PPARγ and Ace-PPARγ expression

To investigate the relationship between P53 and PPARγ after adipogenic differentiation, we performed co-immunoprecipitation and WB, which confirmed that PPARγ interacted with P53 in HEY and MDA-MB-231 PDCs after adipogenic differentiation (**Figure 5C–5F**). *P53* was knocked down in HEY and MDA-MB-231 control cells and PDCs, which were then cultured in adipogenic differentiation medium. Compared with that in cells without *P53* knockdown, the expression of total PPARγ after *P53* knockdown was lower in MDA-MB-231 PDCs with adipogenic differentiation (**Figure 5G**). After *P53* knockdown, Ace-PPARγ expression increased in HEY PDCs and decreased in MDA-MB-231 PDCs undergoing adipogenic differentiation (**Figure 5H and Supplementary Figure S2A**). After *P53* knockdown, FABP4 expression was inhibited in MDA-MB-231 PDCs bearing mutant *P53*, but was promoted in HEY PGCCs and daughter cells bearing wild-type *P53* (**Figure 5I**); the difference in FABP4 expression was statistically significant (for HEY PDCs after adipogenic differentiation: PDCs *vs.* PDCs after *P53* knockdown, *P* < 0.001; for MDA-MB-231 PDCs after adipogenic differentiation: PDCs *vs.* PDCs after *P53* knockdown, *P* < 0.001) (**Figure 5J**). The trend in FABP4 expression was consistent with that of Ace-PPARγ during the adipogenic differentiation of PDCs.

### P300 interacts with P53 and regulates Ace-PPARγ expression in HEY and MDA-MB-231 PDCsafter adipogenic differentiation

**Figure 6:**
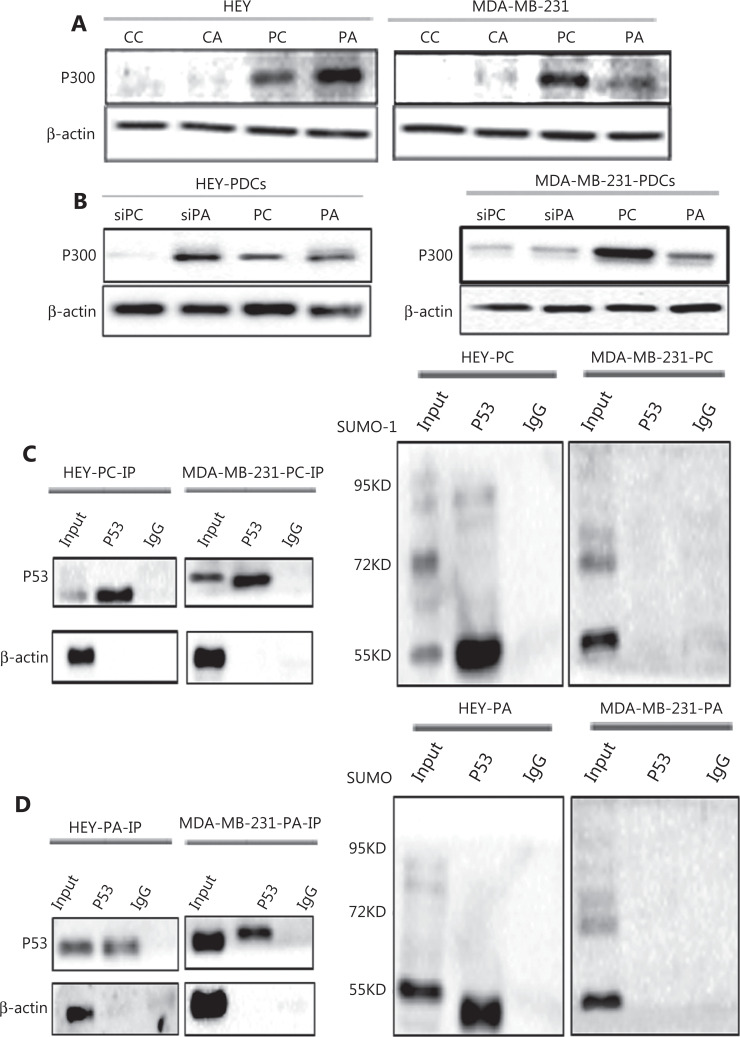

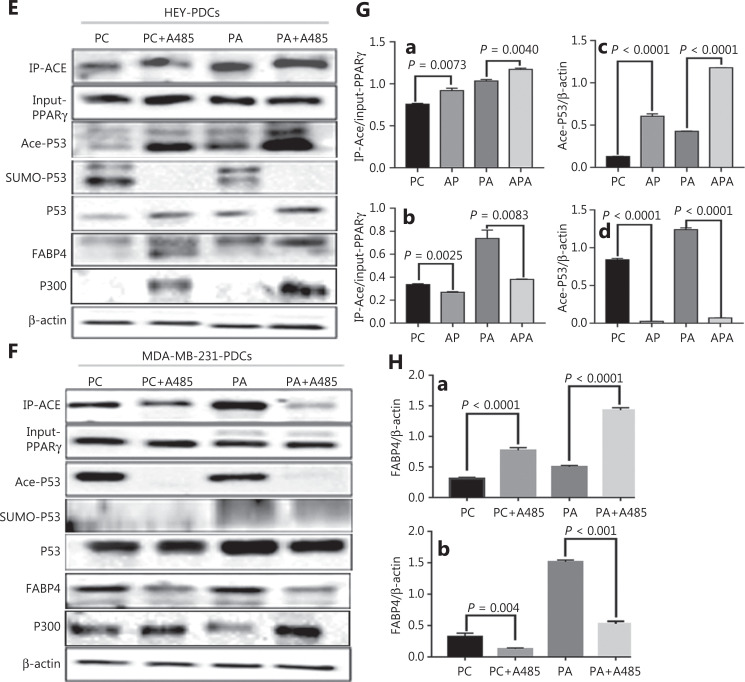
Expression of P300 before and after adipogenic differentiation. (A) Expression of P300 in HEY and MDA-MB-231 control cells and PDCs before and after adipogenic differentiation. (B) Expression of P300 in HEY and MDA-MB-231 PDCs with or without adipogenic differentiation after siRNA-p53 transfection. (C) Total lysates of HEY and MDA-MB-231 PDCs, immunoprecipitated with anti-P53 and immunoblotted with anti-SUMO-1. (D) Total lysates of HEY and MDA-MB-231 PDCs after adipogenic differentiation, immunoprecipitated with anti-P53 and immunoblotted with anti-SUMO-1. (E-F) Expression of Ace-PPARγ, Ace-P53, SUMO-P53, P53, P300, and FABP4 before and after adipogenic differentiation in (E) HEY and (F) MDA-MB-231 PDCs after A485 treatment. (G) Histogram showing the quantitative differences in protein expression in cells after A485 treatment, before and after adipogenic differentiation. (a) Ace-PPARγ expression in HEY PDCs. (b) Ace-PPARγ expression in MDA-MB-231 PDCs. (c) Ace-P53 expression in HEY PDCs. (d) Ace-P53 expression in MDA-MB-231 PDCs. (H) Histogram showing FABP4 expression in HEY (a) and MDA-MB-231 (b) PDCs with or without adipogenic differentiation and A485 treatment.

P300 regulates the ubiquitination-like degradation of P53. Acetylation and ubiquitin-like modification of P53 are competitively performed at the same lysine site^16^. Through immunoprecipitation with anti-P53 and immunoblotting with anti-P300, the interaction between P53 and P300 was detected in the total lysates of HEY and MDA-MB-231 PDCs before and after adipogenic differentiation (**Supplementary Figure S2B**). P53 interacted with P300 in HEY and MDA-MB-231 PDCs after adipogenic differentiation. WB indicated that the expression of P300 increased in HEY PDCs and decreased in MDA-MB-231 PDCs cultured in adipogenic differentiation medium (**Figure 6A**). The P300 expression level further increased in HEY PDCs and decreased in MDA-MB-231 PDCs when *P53* was knocked down (**Figure 6B**).

### SUMOylated P53 regulates adipogenic differentiation of HEY and MDA-MB-231 PDCs

A485 is a potent and selective catalytic inhibitor of P300/CBP^15^, and the acetylation sites in P53 overlap with the ubiquitination or SUMOylation sites^17^. Through IP with anti-P53 and immunoblotting with anti-SUMOylation, we confirmed that P53 is modified by SUMOylation in HEY and PDCs undergoing adipogenic differentiation. SUMOylated P53 was not detected in the MDA-MB-231 PDCs (**Figure 6C and 6D**). The effect of A485 on the viability of HEY and MDA-MB-231 PDCs was analyzed with CCK8 assays. On the basis of these results, 72 h pretreatment with 5 μM A485 was used to inhibit the HAT activity of P300 (**Supplementary Figure S2C**). After A485 treatment, the expression of Ace-P53, Ace-PPARγ, and FABP4 increased, whereas that of SUMOylated P53 decreased in HEY PDCs (**Figure 6E**). The differences in Ace-P53, Ace-PPARγ, and FABP4 were statistically significant (for Ace-PPARγ expression in HEY: PDCs *vs.* PDCs after A485 treatment, *P* = 0.0073; PDCs *vs.* PDCs after adipogenic differentiation and A485 treatment, *P* = 0.0040; for Ace-p53 expression in HEY: PDCs *vs.* PDCs after A485 treatment, *P* < 0.0001; PDCs *vs.* PDCs after adipogenic differentiation and A485 treatment, *P* < 0.0001; FABP4 expression in HEY: PDCs *vs*. PDCs after A485 treatment, *P* < 0.0001; PDCs *vs.* PDCs after adipogenic differentiation and A485 treatment, *P* < 0.001) (**Figure 6Ga, 6Gc and 6Ha**), whereas the expression of Ace-P53, Ace-PPARγ, and FABP4 decreased in MDA-MB-231 PDCs (**Figure 6F**). The differences in Ace-P53, Ace-PPARγ, and FABP4 were statistically significant (for Ace-PPARγ expression in MDA-MB-231: PDCs *vs.* PDCs after A485 treatment, *P* = 0.0025; PDCs *vs.* PDCs after adipogenic differentiation and A485 treatment *P* = 0.0083; for Ace-p53 expression in HEY: PDCs *vs.* PDCs after A485 treatment, *P* < 0.0001; PDCs *vs.* PDCs after adipogenic differentiation and A485 treatment *P* < 0.0001; FABP4 expression in HEY: PDCs *vs*. PDCs after A485 treatment, *P* = 0.004; PDCs *vs.* PDCs after adipogenic differentiation and A485 treatment *P* < 0.0001) (**Figure 6Gb, 6Gd and 6Hb**). SUMOylated P53 was not expressed in MDA-MB-231 PDCs. The P300 histone acetyltransferase activity assays also indicated that A485 treatment inhibited the activity of P300 and increased the expression of Ace-P53 by inhibiting the SUMOylation of P53 in HEY PDCs bearing wild-type *P53*, and decreased the expression of Ace-P53 in MDA-MB-231 PDCs bearing mutant *P53* (**Supplementary Figure S2D**).

### Xenografts from cancer cells with adipogenic differentiation

**Figure 7:**
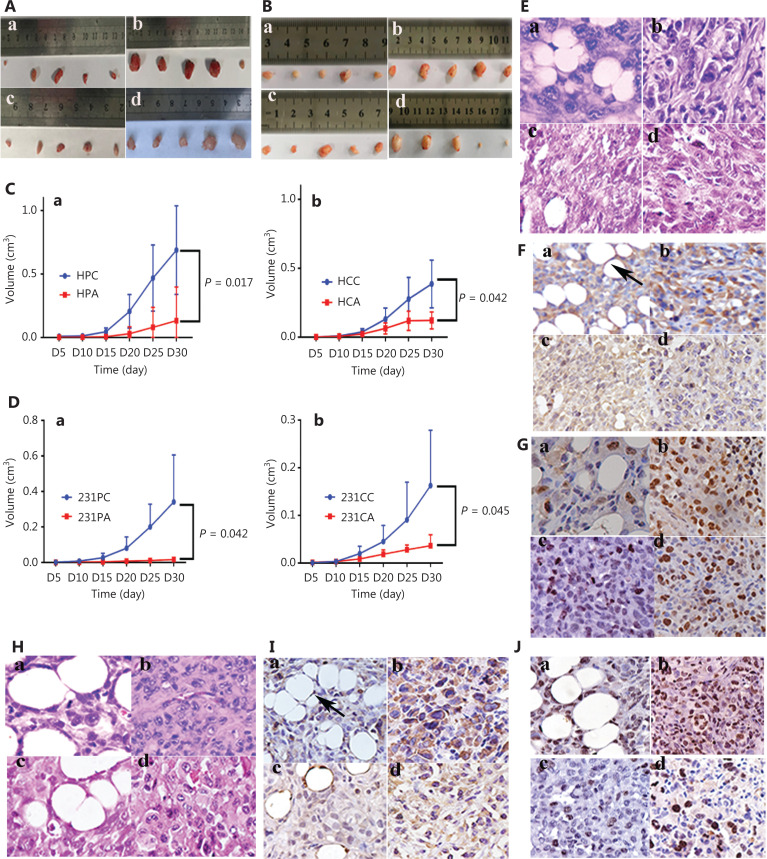

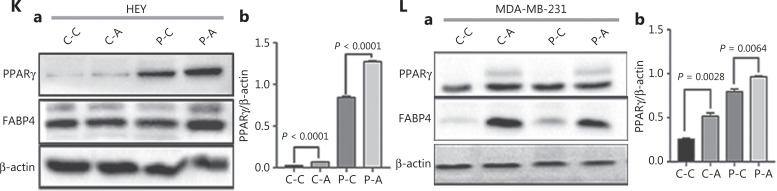
Xenografts from HEY and MDA-MB-231 control cells and PDCs with or without adipogenic differentiation. Tumor masses injected with (A) HEY and (B) MDA-MB-231 (a) PGCCs with their daughter cells after adipogenic differentiation, (b) PDCs, (c) control cells after adipogenic differentiation, and (d) control cells. Tumor growth curves in (C) HEY and (D) MDA-MB-231 (a) PDCs and (b) control cells before and after adipogenic differentiation. (E) H&E staining (×100) of the xenograft tumor injected with HEY (a) PDCs after adipogenic differentiation, (b) PDCs, (c) control cells after adipogenic differentiation, and (d) control cells. (F-G) IHC staining (×100) of (F) human-specific vimentin and (G) Ki-67 in tumors injected with HEY (a) PDCs after adipogenic differentiation (black arrows point the adipocytes), (b) PDCs, (c) control cells after adipogenic differentiation, and (d) control cells. (H) H&E staining (×100) of xenograft tumors injected with MDA-MB-231 (a) PDCs after adipogenic differentiation, (b) PDCs, (c) control cells after adipogenic differentiation, and (d) control cells. (I-J) IHC staining (×100) of (I) vimentin (J) Ki-67 in tumors injected with MDA-MB-231 (a) PDCs after adipogenic differentiation, (b) PDCs, (c) control cells after adipogenic differentiation, and (d) control cells. (K) (a) Western blot showing the expression of PPARγ and FABP4 in xenograft tumor tissues from HEY control cells and PDCs before and after adipogenic differentiation. (b) Statistical graph showing the difference in PPARγ expression. (L) (a) Western blot showing the expression of PPARγ and FABP4 in xenograft tumor tissues from MDA-MB-231 control cells and PDCs before and after adipogenic differentiation. (b) Statistical graph showing the difference in PPARγ expression.

Xenografts were established by subcutaneous injection of HEY (**Figure 7A and Supplementary Figure S2E**) and MDA-MB-231 (**Figure 7B and Supplementary Figure S2F**) control cells and PDCs before and after adipogenic differentiation into BALB/c nude mice. On the 30^th^ day after inoculation, all mice were euthanized, and the tumors were subjected to IHC and WB. Tumor growth curves showed that the average volumes of xenograft tumors inoculated with cells with adipogenic differentiation were significantly smaller than those of tumors inoculated with cells without adipogenic differentiation (**Figure 7C and 7D**). H&E staining was performed to observe morphological characteristics, and IHC staining was used to analyze the expression of human-specific vimentin and Ki-67. As compared with the xenografts from the CC group (**Figure 7Ed and 7Hd**), CA group (**Figure 7Ec and 7Hc**), and PC group (**Figure 7Eb and 7Hb**), more adipocytes were observed in the PDCs of the PA group (**Figure 7Ea and 7Ha**). Adipocytes in the PA group were positive for human-specific vimentin, thus confirming the human origin of the adipocytes (**Figure 7Fa and 7Ia**). Tumor cells in the CC (**Figure 7Fd and 7Id**), CA (**Figure 7Fc and 7Ic**), and PC (**Figure 7Fb and 7Ib**) groups were positive for vimentin, on the basis of IHC staining. In addition, the proportion of Ki-67-positive staining was lower in the PA group (**Figure 7Ga and 7Ja**) than in the CC (**Figure 7Gd and 7Jd**), CA (**Figure 7Gc and 7Jc**) and PC groups (**Figure 7Gb and 7Jb**). WB indicated that the expression levels of PPARγ and FABP4 in xenograft tumor tissues from HEY and MDA-MB-231 control cells and PDCs after adipogenic differentiation were higher than those in cells before adipogenic differentiation (**Figure 7K**), and the differences were statistically significant (for HEY: control *vs.* control after adipogenic differentiation, *P* < 0.0001; PDCs *vs.* PDCs after adipogenic differentiation *P* < 0.0001; for MDA-MB-231: control *vs.* control after adipogenic differentiation, *P* = 0.0028; PDCs *vs.* PDCs after adipogenic differentiation *P* = 0.0064) (**Figure 7L**).

## Discussion

Differentiation therapy has long been recognized in the treatment of malignant tumors, and cancer cells with high plasticity can differentiate into more mature tumor cells; therefore, this therapy may have potential for highly malignant tumors. NCI-H446 cells can be induced to differentiate into neurons, adipocytes, and bone cells *in vitro*^18^. Cancer cells with homologous recombination defects, such as ovarian and breast cancer cells with BRCA1/2 mutations, can be induced to differentiate with poly ADP-ribose polymerase inhibitors^19^. Thyroid cancer cells expressing the PPARγ fusion protein can be induced to differentiate into adipocyte-like cells with pioglitazone^20^. Gupta et al.^21^ have identified that the potassium ionophore salinomycin induces epithelial differentiation of tumor cells and inhibits tumor growth in human breast cancer. Cancer cell plasticity facilitates the development of therapeutic resistance and the progression of malignancy. EMT enhances cellular plasticity and can be exploited therapeutically by forcing the transdifferentiation of EMT-derived cancer cells into functional cells^3^. Daughter cells derived from PGCCs undergo EMT and exhibit strong plasticity^22,23^. In this study, we confirmed that HEY and MDA-MB-231 PDCs undergo EMT, thus obtaining a mesenchymal cell phenotype and stem cell characteristics, and can differentiate into adipocytes. The migration, invasion, and proliferation abilities of PDCs decreased, and 2 cell cycle rounds were observed in HEY and MDA-MB-231 PDCs after adipogenic differentiation. The transformation between cell proliferation and adipogenic differentiation is regulated by the cell cycle and differentiation factors^24^. Growth-arrested preadipocytes undergo several cell cycle rounds before terminally differentiating into adipocytes, thus suggesting that crosstalk exists between the cell cycle and cell proliferation.

PPARγ is a critical regulator of adipogenic differentiation^23^. Protein phosphorylation is a type of PTM that regulates a wide range of signaling pathways involved in differentiation, apoptosis, proliferation, gene regulation, and metabolism^25–27^. PPARγ is a short-lived protein^28^ that is regulated by a series of PTMs^29^, including phosphorylation and acetylation^30^. PPARγ can be phosphorylated by mitogen-activated protein kinases, cyclin-dependent kinase 5, and AMP-activated protein kinase. The phosphorylation of PPARγ decreases the expression of PPARγ mRNA and protein, and inhibits adipogenic differentiation^31,32^. The deacetylation of PPARγ by SIRT1 downregulates the expression of PPARγ and inhibits adipogenic differentiation^24^. The acetylation of the conserved lysine motif (K154/155) of PPARγ1 promotes lipid synthesis in ErbB2-positive breast cancer cells^33^. Our study confirmed that the expression levels of PPARγ and FABP4 gradually increased, and that of phospho-PPARγ (Ser112) decreased in HEY and MDA-MB-231 PDCs with increasing culture time in adipogenic differentiation medium. Helenius et al.^34^ have confirmed that phosphorylation inhibits the ability of PPARγ to promote adipogenic differentiation. Phosphorylation of PPARγ decreases its transcriptional activity, promotes ubiquitination, and further limits its ability to act as a transcriptional activator^35^.

The deacetylation of PPARγ by SIRT1 downregulates PPARγ expression and inhibits adipogenic differentiation^36^. When PPARγ1 is acetylated at the conserved lysine motif (K154/155), it promotes lipid synthesis in ErbB2-positive breast cancer cells^33^. In this study, we also showed that the expression of Ace-PPARγ was higher in HEY and MDA-MB-231 PDCs after adipogenic differentiation than in PDCs before adipogenic differentiation. The tumor suppressor gene *P53* is involved in cell cycle control, apoptosis, and genomic stability, and *P53* mutations appear in many cancers. The dysregulated expression or function of pRB or P53 is a hallmark of all cancers^37^. Although *P53* is one of the most well-studied genes, its role in adipocytes remains poorly understood. Regulation of PPARγ expression by P53 depends on the *P53* genotype. Our results confirmed that *P53* knockdown in HEY PDCs expressing wild-type *P53* increased Ace-PPARγ expression and facilitated adipogenic differentiation. In MDA-MB-231 PDCs with mutant *P53*, *P53* knockdown inhibited adipogenic differentiation. P300 is a HAT transcriptional coactivator that is critical in several cellular processes^38^. P300-P53 regulates the acetylation level of PPARγ, and silencing P53 or P300 disrupts the formation of the P53-P300 complex^39–41^. In this research, the expression levels of P300 and Ace-PPARγ were associated with the expression of P53 in HEY and MDA-MB-231 cells with different *p53* genotypes. The acetylation sites of P53 overlap with the ubiquitylation or SUMOylation sites^40,41^. We confirmed that P53 was modified by SUMOylation in HEY control cells and PDCs. In HEY PDCs with wild-type *P53*, A485 treatment inhibited the activity of P300 and increased the expression of Ace-P53 by inhibiting SUMOylation of P53. In MDA-MB-231 PDCs with mutant *P53*, A485 treatment decreased the activity of P300. The decreased activity of P300 inhibited the acetylation of P53, thereby decreasing adipogenic differentiation.

## Conclusions

**Figure 8:**
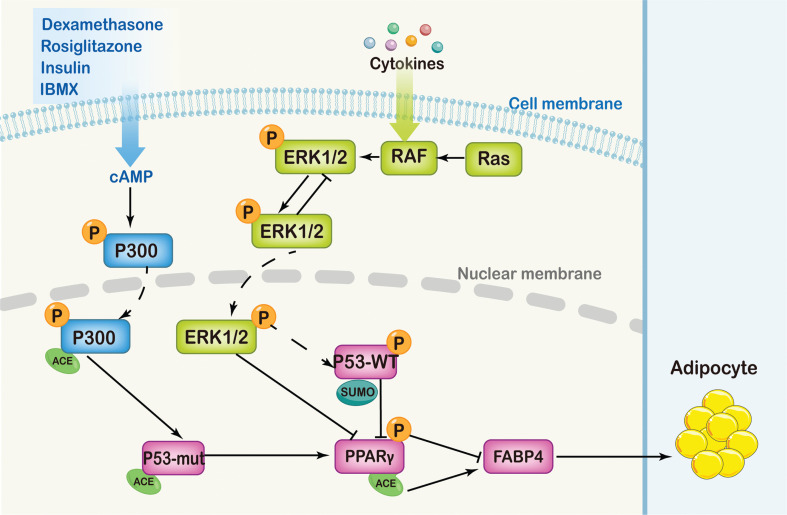
The relevant mechanism of PDCs differentiated into adipocytes. Dexamethasone, rosiglitazone, insulin, and IBMX are all potent activators of the 3′,5′-cyclic AMP (cAMP)-dependent protein kinase pathway, and cAMP regulates the phosphorylation of P300. P300 is a histone acetyltransferase transcriptional coactivator that mediates the acetylation of P53. Acetylated P53 is in an activated state, and positive feedback regulates P300. P300-P53 interacts with PPARγ and regulates the acetylation level of PPARγ. Mutant P53 is acetylated and modified by P300, and has a beneficial function in the adipogenic differentiation of PDCs, whereas wild-type *P53* is ubiquitinated by P300, thus negatively regulating the adipogenic differentiation of PDCs. As a member of the nuclear-receptor superfamily, PPARγ induces growth arrest and adipogenic differentiation. In addition, the RAS/RAF/ERK signaling pathway is associated with the development and progression of malignant tumors. Activated ERK1/2 leads to PPARγ phosphorylation, which in turn inhibits adipocyte differentiation. FABP4, a member of the FABP family, is abundantly expressed in adipocytes and serves as a marker of successful adipogenic differentiation. The expression of FABP4 depends on the different post-translational modifications of PPARγ. During adipogenic differentiation of PGCs, the phosphorylation level of PPARγ gradually decreases. The increased acetylation level of PPARγ in PDCs promotes lipid synthesis.

Acetylation of P53 and PPARγ plays an important role in regulating adipogenic differentiation in PDCs. The high cellular plasticity of PDCs may be exploited therapeutically for the transdifferentiation of cancer cells with strong cell plasticity into post-mitotic and functional mature tumor cells. In this study, we showed that *P53* genotypes and Ace-PPARγ expression were associated with the adipogenic differentiation of PDCs. However, other PTMs (phosphorylation and SUMOylation) of critical regulators in the process through which cancer cells with high plasticity are induced to differentiate into adipocytes must be studied in the future (**Figure 8**). Additional research on the molecular mechanisms of differentiation therapy may provide new therapeutic strategies for the treatment of highly malignant tumors.
