# Supplementary material for: Acetylated-PPARγ expression is regulated by different P53 genotypes associated with the adipogenic differentiation of polyploid giant cancer cells with daughter cells
Source: Cancer Biol Med. 2023 Jan 12;20(1):56–76. doi: 10.20892/j.issn.2095-3941.2022.0432 (PMC9843444; doi:10.20892/j.issn.2095-3941.2022.0432)
Supplement: Supplementary file 1 [file cbm-20-056-s001.pdf]

Supplementary materials

Table S1 Antibodies used in this study

Reagent	Species specificity	Company	WB	ICC	IHC
PPARγ	Mouse monoclonal	Abcam	1:1,000	1:500	
FABP4	Rabbit polyclonal	Affinity	1:1,000	1:200	1:200
ACE-Lys	Rabbit polyclonal	CST	1:1,000		
Phospho-PPARγ (Ser112)	Rabbit polyclonal	bioss	1:300		
P53	Rabbit monoclonal	Proteintech	1:1,000		
Acetyl-P53 (Lys382)	Rabbit polyclonal	CST	1:1,000		
SUMO-1	Rabbit polyclonal	CST	1:1,000		
P300	Rabbit monoclonal	Proteintech	1:1,000		
Vimentin	Rabbit monoclonal	Abcam			1:200
Ki-67	Rabbit monoclonal	Abcam			1:200
β-actin	Mouse monoclonal	Proteintech	1:1,000		
GAPDH	Mouse monoclonal	Proteintech	1:3,000		

Table S2 *p53*-siRNA interfering sequences

Names	Sense (5´-3´)	Antisense (5´-3´)
*p53*i-339	CCGGACGAUAUUGAACAAUTT	AUUGUUCAAUAUCGUCCGGTT
*p53*i-886	GUACCACCAUCCACUACAATT	UUGUAGUGGAUGGUGGUACTT
*p53*i-985	GUAAUCUACUGGGACGGAATT	UUCCGUCCCAGUAGAUUACTT
*p53*i-GAPDH	UGACCUCAACUACAUGGUUTT	AACCAUGUAGUUGAGGUCATT
*p53*i-NC	UUCUCCGAACGUGUCACGUTT	ACGUGACACGUUCGGAGAATT

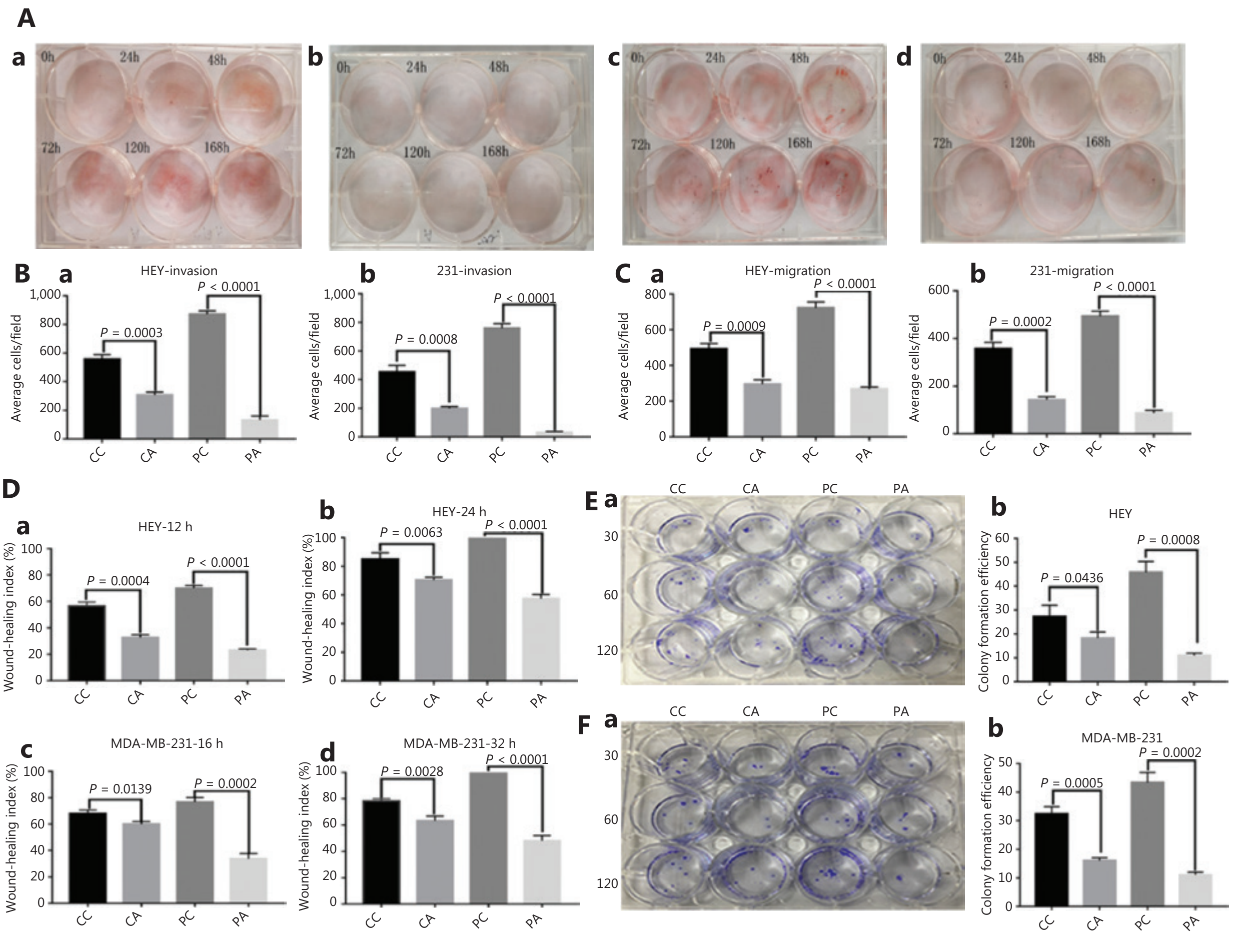

Figure S1 Oil red O (ORO)-staining, and migration, invasion, and proliferation activities in HEY and MDA-MB-231 control cells and PGCCs with daughter cells with or without adipogenic differentiation. (A) ORO staining, showing the adipogenic differentiation in (a) HEY PGCCs with daughter cells, (b) HEY control cells, (c) MDA-MB-231 PGCCs with daughter cells, and (d) MDA-MB-231 control cells, after treatment with adipogenic differentiation medium at 0, 24, 48, 72, 120, and 168 h. (B) Column diagram showing the invasion efficiency in (a) HEY and (b) MDA-MB-231 control cells and PGCCs with daughter cells before and after adipogenic differentiation. (C) Column diagram showing the migration efficiency in (a) HEY and (b) MDA-MB-231 control cells and PGCCs with daughter cells before and after adipogenic differentiation. (D) Wound-healing index in HEY and MDA-MB-231 control cells and PGCCs with daughter cells before and after differentiation. (E) Plate clone formation experiment comparison of (a) the proliferation ability in HEY control cells and PGCCs with daughter cells before and after differentiation. (b) Column diagram showing the colony formation efficiency between 30, 60, and 120 cells. (F) Plate clone formation experiment comparison of (a) the proliferation activity in MDA-MB-231 control cells and PGCCs with daughter cells before and after differentiation. (b) Column diagram showing the colony formation efficiency between 30, 60, and 120 cells.

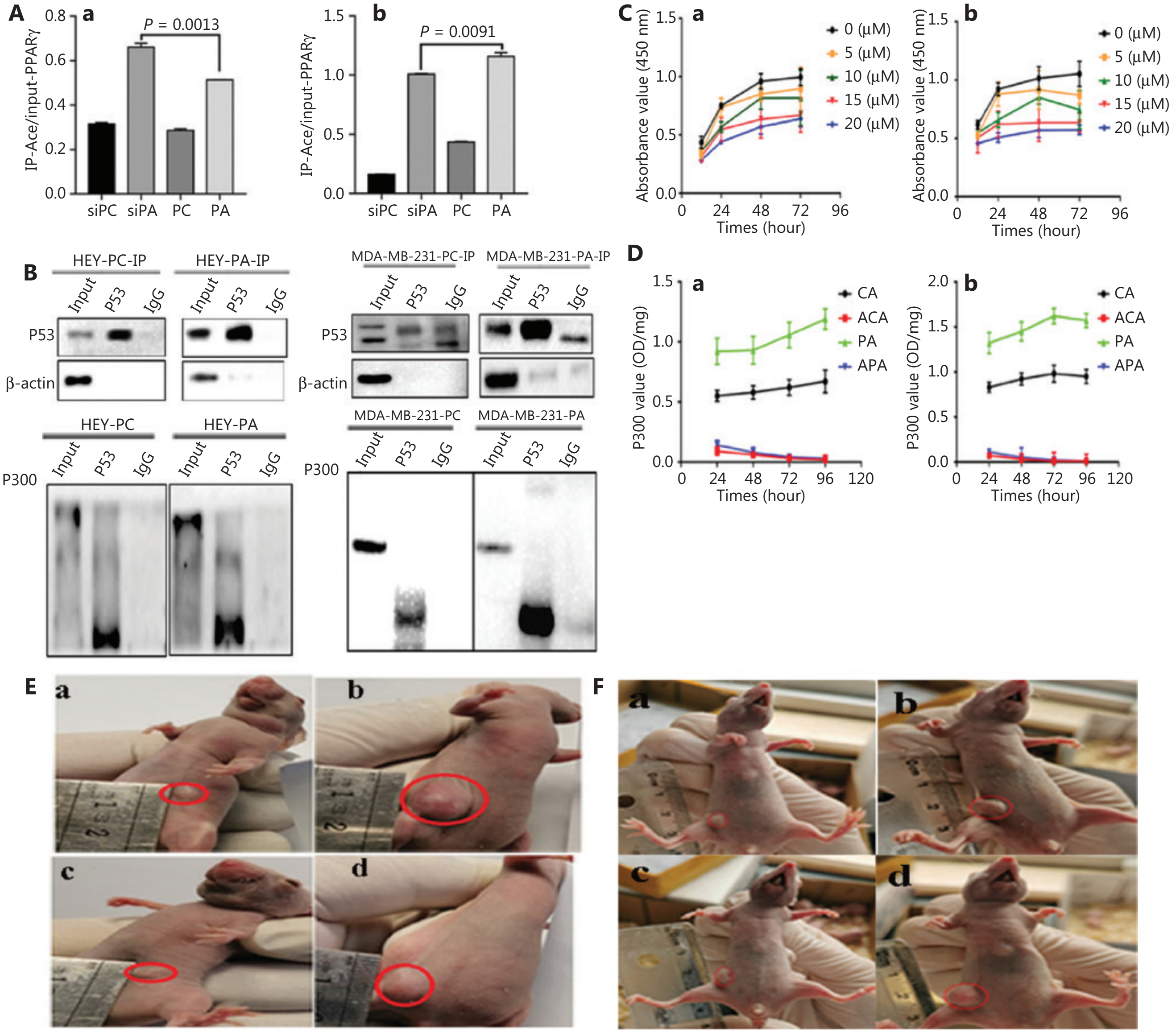

Figure S2 (A) Column diagram showing the Ace-PPARγ expression in HEY (a) and MDA-MB-231 (b) PGCCs with daughter cells undergoing adipogenic differentiation after *p53* knockdown. (B) Total lysates of HEY and MDA-MB-231 PGCCs with daughter cells before and after adipogenic differentiation, immunoprecipitated with anti-P53 and immunoblotted with anti-P300. (C) The influence of different concentrations of A485 on the cell viability of (a) HEY PGCCs with daughter cells and (b) MDA-MB-231 PGCCs with daughter cells, detected with CCK8 assays. (D) A485 was used to inhibit the HAT activity of P300 in HEY (a) and MDA-MB-231 (b) PGCCs with daughter cells after adipogenic differentiation. (E) Gross pictures of xenografts injected with HEY (a) PDCs after adipogenic differentiation, (b) PDCs, (c) control cells after adipogenic differentiation, and (d) control cells. (F) Gross pictures of xenografts injected with MDA-MB-231 (a) PDCs after adipogenic differentiation, (b) PDCs, (c) control cells after adipogenic differentiation, and (d) control cells.
